# Preventive Effect on Seroma of Use of PEAK PlasmaBlade after Latissimus Dorsi Breast Reconstruction

**DOI:** 10.1097/GOX.0000000000002035

**Published:** 2018-12-17

**Authors:** Yoshihiro Sowa, Naoki Inafuku, Takuya Kodama, Daiki Morita, Toshiaki Numajiri

**Affiliations:** From the Department of Plastic and Reconstructive Surgery, Kyoto Prefectural University of Medicine, Graduate School of Medical Sciences.

## Abstract

Postoperative seroma is still the main complication after a latissimus dorsi (LD) flap procedure. The etiology of seroma is currently thought to comprise tissue fluids resulting from inflammatory reactions in affected tissue caused by the use of monopolar electrocautery (EC). It is possible that seroma formation can be reduced by using alternative devices such as the PEAK PlasmaBlade (PPB), which provides atraumatic scalpel-like cutting precision while the blade temperature remains close to body temperature. The subjects were 44 patients who underwent breast reconstruction with LD flaps from August 2015 to April 2017. They were retrospectively split into groups treated with a PPB (n = 21) and with conventional EC (n = 23). Outcomes such as rate of seroma formation, total drain discharge volume, indwelling period of drainage at the donor site, length of hospital stay, and operation time were compared between the 2 groups. The incidence of seroma was significantly lower in the PPB group (19.0%) than in the EC group (47.8%). The total drain discharge volume was significantly lower and the indwelling period of drainage and length of hospital stay were significantly shorter in the PPB group. In summary, use of PPB in an LD flap procedure can reduce seroma formation and the lengths of the drainage period and the hospital stay.

## INTRODUCTION

The latissimus dorsi (LD) flap is a common and useful tool for breast reconstruction that has many advantages, including safety and ease of use. However, seroma at the donor site is still the main complication after an LD flap procedure.^1–3^ A seroma is now thought to mainly comprise tissue fluids resulting from disruption of lymphatic vessels and inflammatory reactions in affected tissue caused by use of monopolar electrocautery (EC).^1–5^ We previously reported that use of Harmonic Focus shears (HFS) for LD flap elevation, instead of EC, reduced the lengths of the drainage period and hospital stay after surgery.^3^ However, we could not find evidence that HFS significantly reduced the incidence of seroma formation, and HFS also has a high cost.

**Fig. 1.**
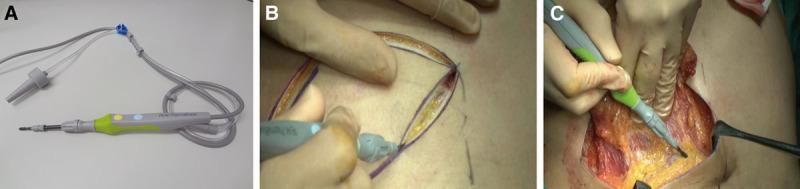
The electrosurgical device and intraoperative pictures. A, The PPB (Medtronic Inc., Minneapolis, Minn.). Precise pulses of RF energy and proprietary insulation material on both sides of the blade enable the devise to dissect like a scalpel and control bleeding, while causing minimal thermal damage to surrounding tissue. B, Intraoperative image of skin incision. C, The dissection surface of the LD muscle using the PPB. RF, radiofrequency.

The PEAK PlasmaBlade (PPB) is a new electrosurgical device that has lower cost and provides more atraumatic, scalpel-like cutting precision on skin, while the blade temperature stays close to body temperature and provides electrosurgical-like hemostasis, resulting in minimal tissue injury (Fig. 1).^6,7^ Therefore, there is a possibility that seroma formation can be significantly reduced using the PPB. The aim of this study was to evaluate the benefits of the PPB relative to conventional EC for tissue dissection, in terms of operation time, length of hospital stay, and complications, and to investigate the efficacy of the PPB for reduction and prevention of seroma in an LD flap procedure.

## METHODS

A retrospective study was conducted in 44 patients who underwent breast reconstruction with LD flaps at Kyoto Prefectural University Hospital. Patients were classified into groups based on the device used for tissue incision and dissection for elevation of the LD flap: 21 patients treated with a PPB (Medtronic Inc., Minneapolis, Minn.) between February 2016 and December 2017, and 23 patients treated using conventional EC between August 2015 and February 2016. Medical charts were reviewed to obtain information mainly on age, body mass index, flap weight, rate of seroma, drain reinsertion, drainage volume, indwelling period of drainage, hospital stay, and operation time, volume of bleeding for each patient. The outcomes were the rate of seroma formation, total drain discharge volume, indwelling period of drainage at the donor site, length of hospital stay, and operation time. In all patients, 15Fr vacuum drainage tubes were placed below the transplanted LD flap and donor site at completion of the surgical procedure. The drains were removed when drainage was < 30 mL/24 h or at 2 weeks postoperatively. After drain removal, puncture drainage with a needle was performed once a week if liquid accumulated at the donor site. Seroma was defined as the persistence of seroma for more than 4 weeks postoperatively. Statistical analysis was performed with JMP (SAS Institute, Cary, N.C.) using an independent *t* test, Mann-Whitney *U* test, and a Pearson chi-square test for comparison between the groups.

## RESULTS

Patient characteristics are shown in Table 1. The incidence of seroma was significantly lower in the PPB group than in the EC group (*P* < 0.043). The total drain discharge volume was also significantly lower in the PPB group. Use of a PPB reduced the median indwelling period of drainage at the donor site from 9.43 days to 7.91 days, and this may have significantly led to a similar decrease of 2 days in the length of hospital stay in the PPB group (*P* < 0.011). A postoperative bleeding at the donor site occurred in the PPB group (Table 2).

**Table 1.**
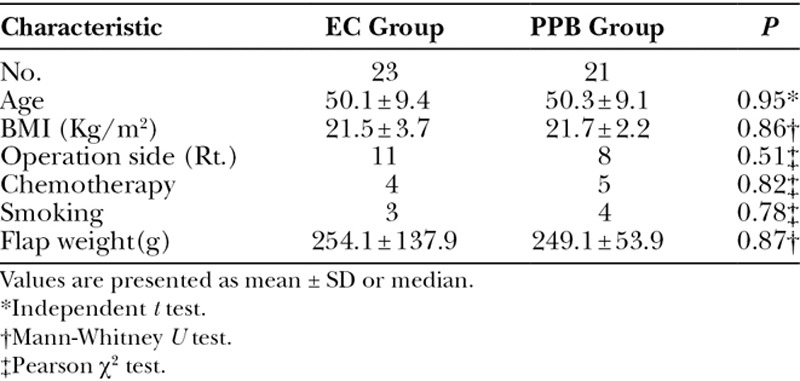
Patient Demographics

**Table 2.**
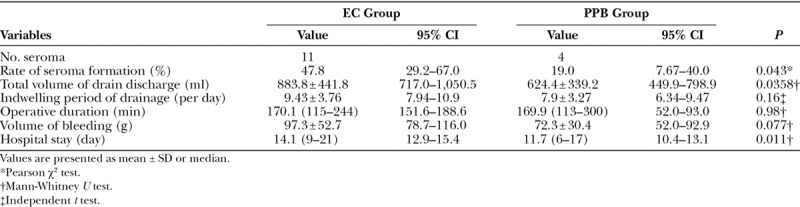
Results for Outcome Measures

## DISCUSSION

**Fig. 2.**
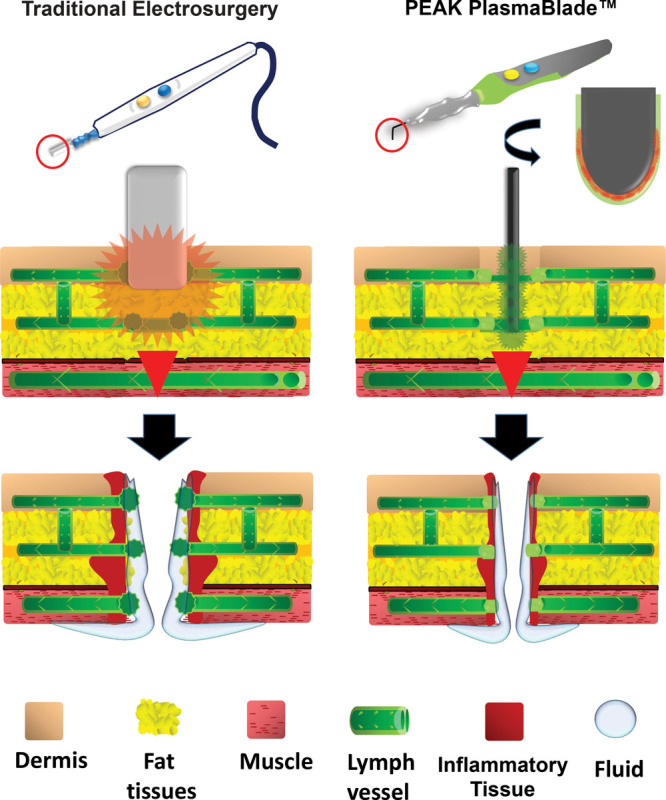
Schematic illustration shows the speculation suggesting that seroma formation is a consequence of surgical disruption of lymph vessels, with ensuing leakage of fluid and inflammatory exudates into the dead space created by the surgical dissection. There is a possibility that seroma formation can be prevented or reduced by using alternative devices like PPB that can dissect the tissue with minimal thermal damage to surrounding tissue.

The PPB is a soft-tissue dissection device that uses brief, precise pulses of radiofrequency energy that, combined with a proprietary insulation technology, enables electrosurgical dissection with the precision of a scalpel and the bleeding control of traditional electrosurgery, while producing minimal thermal damage to surrounding tissue (Fig. 2).^6–8^ Some studies have shown that low thermal injury technology offers some benefits compared with use of a cold scalpel and EC.^6,7^ In a clinical study, Dogan et al.^9^ found that use of a PPB in breast surgery significantly reduced the total volume of drain discharge and the indwelling period, compared with use of EC.

In this study, we evaluated the PPB using an experimental design adapted from our previous assessment of the effect of HFS on reducing the frequency of seroma after an LD flap procedure. We found that the PPB significantly reduced the rate of seroma, whereas HFS did not have this effect. Furthermore, our results suggest that use of a PPB shortens the indwelling period of drainage and the hospital stay, as also found with HFS. Di Monta et al.^10^ have shown that a collagen sealant patch reduces lymphatic drainage after lymph node dissection, which indicate that the etiology of seroma might be disruption of lymphatic vessels and inflammatory reactions.

Both devices can potentially prevent seroma, but the mechanisms may be somewhat different. The PPB permits incision and dissection with minimal thermal damage to surrounding tissue, which might be more effective for reducing the acute inflammatory response, whereas HFS might be superior at closing the small vascular and lymphatic vessels created by hydrogen bond degradation. It is difficult to define the superiority or inferiority of PPB and HFS, but PPB is particularly useful because it can be used for all processes required in elevation of the LD flap, including skin incision, and it has a lower cost. However, we experienced 1 PPB-treated case with postoperative bleeding at the donor site, which suggests that care must be taken to avoid such events in intraoperative hemostasis. This study is the first to examine pulsed plasma-mediated electrosurgical technology for LD flap elevation. Our results suggest positive outcomes, but the study has limitations of a retrospective design and a relatively small sample size. Therefore, generalization of the results requires performance of further well-controlled studies.

In summary, this study demonstrated that use of PPB in an LD flap procedure can reduce seroma formation and the lengths of the drainage period and the hospital stay.
